# Fabrication of a tunable mesoporous polypyrrole/MXene composite with a sandwich structure for enhancing electromagnetic wave absorption performance†

**DOI:** 10.1039/d5ra00972c

**Published:** 2025-04-03

**Authors:** Wenjuan Zhang, Xiangyue Yang, Youliang Wang, Yaxian Wang, Xuyang Wu, Yongqian Shen

**Affiliations:** a State Key Laboratory of Advanced Processing and Recycling of Non-ferrous Metals, Lanzhou University of Technology Lanzhou 730050 China wenjuanzhang86@163.com; b School of Mechanical and Electrical Engineering, Lanzhou University of Technology Gansu Lanzhou 730050 People's Republic of China wangyouliang20@163.com

## Abstract

As wireless communication technologies and electronic devices continue to advance quickly, electromagnetic waves (EMWs) have a serious impact on equipment interference and human health. The development of new materials for EMW absorption and shielding is crucial to mitigate electromagnetic (EM) interference. In this study, a unique strategy was implemented for developing a mesoporous polypyrrole/MXene (mPM) composite. The mPM composite was prepared by a molecular synergistic self-assembly method using spherical block copolymer micelles as templating agents to regulate mesopores. The effects of the polypyrrole/MXene ratio and pore size on the EMWs absorption performance of composites were systematically investigated. The mPM composite formed by MXene and polypyrrole exhibits a well-developed porous structure and conductive network, significantly enhancing dielectric loss. The rich mesoporous structures and the multi-layer heterogeneous interfaces can improve the interface polarization, realize multiple reflections, and enhance the EMWs absorption performance of the mPM. The mPM-1 composite, prepared using the PS_100_-*b*-PEO_114_ template, achieved a reflection loss (RL) of −67.82 dB at an MXene to polypyrrole ratio of 1 with a matching thickness of 1.37 mm. Its effective absorption bandwidth (EBW) was 3.68 GHz (ranging from 14.16 to 17.84 GHz) with a matching thickness of 1.25 mm. It is a reliable way to develop absorbing materials with light, thin, large EBW and high-quality EMWs absorption performance.

## Abstract 2

A mesoporous polypyrrole/MXene (mPM) composite with multi-layer sandwich-like structures was developed *via* molecular self-assembly templated by block copolymer micelles, enhancing dielectric loss for superior electromagnetic wave absorption.

## Graphical abstract

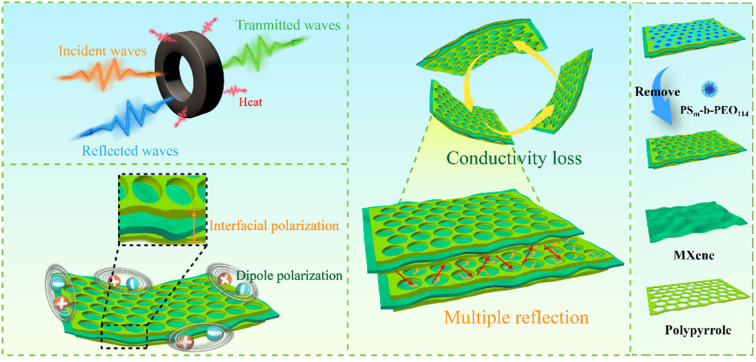

## 1. Introduction

With the advancement and widespread use of wireless communication technology, electromagnetic waves (EMWs) have been used as carriers in a variety of industries in recent years, and greatly improve human productivity and quality of life. However, the electromagnetic (EM) interference is one of the most serious threats to both the environment and human health.^1,2^ Therefore, microwave-absorbing materials are increasingly essential in mitigating the effects of EMW pollution and safeguarding human health.^3–7^ In addition, controlling the loss and interference of EMWs is critical to meeting the growing demands in military applications.^8,9^

At present, metallic materials^10,11^ are widely used to suppress EM pollution due to their high electrical conductivity, but their high density, difficult processing, and weak corrosion resistance limit their application in highly integrated electronic devices.^12^ The ongoing focus on EM interference and radiation has prompted the development of new low-density, effective microwave-absorbing materials in recent years.^13–15^ The emergence of nanomaterials such as nanoparticles,^16,17^ nanowires,^18,19^ porous carbon,^20,21^ graphene,^22,23^ and transition metal carbides, nitrides, or carbonitrides^24^ has brought new perspectives to EM protection systems. Among these materials, MXene is particularly notable for its rich surface functional groups and high electrical conductivity,^25^ which are advantageous for EMW attenuation and absorption, making it a highly promising candidate for microwave absorption applications. The inhomogeneous interfaces on the surface of MXene produce defect polarization, interfacial polarization, and multiple reflections between the interfaces, which enhance the microwave attenuation ability and broaden the absorption bandwidth.^26^ MXene compounds with other materials through hydrogen bonding can accelerate the electron transfer and improve the microwave absorption performance. However, the high electrical conductivity and strong dielectric loss of MXene can adversely affect its impedance matching, limiting its performance as a high-efficiency microwave-absorbing material. Generally, the utilization of high-conductivity composites to enhance conductivity and adjust impedance matching is an effective method for improving the microwave absorption properties of MXene-based composites.

Polypyrrole (PPy) is a crucial conductive polymer material. Owing to its simple synthesis process, low density, excellent conductivity, robust corrosion resistance, and stable chemical properties, it has attracted increasing attention in the field of microwave absorption.^27^ It is reported that various PPy-based composites exhibit excellent microwave absorbing properties.^28^ Lei *et al.*^29^ employed an *in situ* polymerization method to coat PPy particles onto layered Ti_3_C_2_T_*x*_ MXene. The heterogeneous interfaces and the synergistic effects between the layered MXene and the conductive PPy network effectively enhance the microwave absorption performance. The optimal reflection loss reaches −68 dB at 2.68 mm thickness; at the same time, the EBW reaches 6.56 GHz (from 11.44 to 18 GHz) when the thickness is 2.26 mm, covering the entire Ku band (12–18 GHz). As a result, MXene/polymer-based materials possess advantages such as interface polarization, dipole polarization, and conductivity, which facilitate dielectric loss and enhance impedance matching.

In addition, conductive polymers have the characteristics of easy morphological regulation and resistance to oxidation. Based on this, compositing the conductive polymer PPy with MXene not only introduces multiple loss mechanisms but also effectively protects the MXene layer from oxidation, providing an effective approach to obtaining ideal absorbing materials. In addition, the structure of absorbing materials can also modulate their microwave absorption performance. Among these materials, porous materials have shown great potential for the development of absorbing materials due to their special pore structure, which provides excellent electrical conductivity and high EMWs attenuation.^30,31^ However, controlling the pore structure of two-dimensional (2D) porous polymers remains a significant challenge. To date, precise control of pore structure primarily relies on two methods: the template method and molecular self-assembly. The template method is mainly to crosslink the monomer on the surface of a pre-prepared 2D silica-based template, followed by etching the template with corrosive acids or alkalis to obtain the porous structure. Although this method can synthesize a variety of 2D porous polymers, its pore structure is constrained by the morphology of the template, resulting primarily in spherical mesopores. Additionally, this method is complex, costly, and unsuitable for large-scale production, which limits its practical applications. By contrast, the molecular self-assembly method, which relies on the synergistic organization of polymer precursor and surfactant template on a 2D substrate, is a more effective strategy. Therefore, self-assembled block copolymers offer an excellent route for the construction of intricate mesoporous structures due to the notable advantages of the molecular self-assembly method in terms of predictable production, accurate thickness control, and simple removal without residual.^32–35^ However, the design and synthesis of 2D porous PPy/MXene composites remain significant challenges. The influence mechanism of pore structure on microwave absorption enhancement needs further research.

In this work, a mesoporous polypyrrole/MXene (mPM) composite with well-developed conductive networks was constructed by utilizing the non-homogeneous interface of MXene and the conductivity of the polymer PPy. Meanwhile, the molecular synergistic self-assembly strategy was used to regulate the mesoporous size of the composite by controlling the degree of polymerization of the template agent. The effects of different components and structures on microwave absorption properties were systematically studied by using a vector network analyzer, and the microwave absorption mechanism was clarified.

## 2. Experimental

### 2.1 Materials

Ti_3_AlC_2_ (400 mesh) was obtained from Jilin 11 Technology Co., Ltd, China. PS_50_-*b*-PEO_114_, PS_100_-*b*-PEO_114_, and PS_150_-*b*-PEO_114_ were purchased from Xi'an Ruixi Biological Technology Co., Ltd, China. Hydrochloric acid (HCl) was purchased from Foshan Huaxisheng Chemical Co., Ltd, China. Lithium fluoride (LiF) was purchased from Shanghai Aladdin Biochemical Technology Co., Ltd, China. Potassium bromide (KBr) was purchased from Tianjin Guangfu Technology Development Co., Ltd, China. Lithium chloride (LiCl), pyrrole (Py), and ammonium persulphate (APS) were purchased from Shanghai McLean Biochemical Technology Co., Ltd, China. Tetrahydrofuran (THF) was provided by Tianjin Baishi Chemical Co., Ltd, China. All reagents used in the experiments were of analytical grade. Deionized water (DI water) was used throughout all experiments.

### 2.2 Preparation of delaminated MXene (DL-MXene)

The Ti_3_C_2_ MXene was successfully synthesized through selective etching of the Al layer from Ti_3_AlC_2_ using the *in situ* HF method. Firstly, a premixed solution (20 mL) of LiF (3 M) and HCl (12 M) was prepared, then it was added with Ti_3_AlC_2_ (1 g), and the mixture was continuously stirred for 48 h at 38 °C. Afterward, the mixture was centrifuged and washed with HCl (1 M), LiCl (1 M), and DI water until the pH of the supernatant exceeded 6. Secondly, the product obtained after freeze-drying was multilayer MXene (ML-MXene). ML-MXene was added to an appropriate volume of DI water and the mixture was sonicated for 45 minutes in a nitrogen (N_2_) atmosphere. The solution was then centrifuged at 8000 rpm for 5 minutes to collect the supernatant, which was subsequently freeze-dried to obtain DL-MXene.

### 2.3 Self-assembly of mPM

Firstly, PS_100_-*b*-PEO_114_ block copolymer (0.3 g) was dissolved in THF (6 mL), and then DI water (6 mL) was slowly added dropwise to the solution to form spherical micelles. Secondly, DI water (42 mL) was added rapidly dropwise to the solution. After stirring for 30 min, different volumes of DL-MXene (2 mg mL^−1^) solution were added, followed by the addition of pyrrole (Py) monomer (192 μL), and the mixture was stirred evenly in an ice bath. Thirdly, the polymerization was initiated by introducing APS solution (10 mg mL^−1^) into the above solution at the ratio of APS/Py = 1 : 1 (molar ratio), and HCl was added to the solution for acid doping until the system reached 1 M HCl, and the system was stirred slowly for 6 h at 0–2 °C. When the reaction was finished, the product was washed with THF, EtOH, and deionized water to pH = 6, and then freeze-dried for 24 h. To study the effect of the ratio of MXene to PPy on the microwave absorption performance, the mass ratios of the MXene to Py were 0.5, 1, 1.5, and 2, which were tagged as mPM-0.5, mPM-1, mPM-1.5, and mPM-2, respectively.

In the above process, the pore size can be adjusted by using different block copolymers, PS_50_-*b*-PEO_114_ and PS_150_-*b*-PEO_114_, and the mass ratio of MXene to Py can be controlled at 1 to obtain the porous MXene/PPy composite with different pore sizes, which are designated as mPM-50 and mPM-150, respectively.

### 2.4 Characterization

The morphology was carried out by a Scanning Electron Microscope (SEM) and a Transmission Electron Microscope (TEM). X-ray diffraction (XRD) images were obtained *via* an X-ray diffraction (scanning range: 5–90°, scanning speed: 5° min^−1^). The magnetic properties were characterized by a Vibration Sample Magnetometer (VSM). Infrared (IR) spectra were obtained by a Fourier Transform Infrared Spectroscopy (FT-IR). The obtained structure was determined *via* X-ray photoelectron spectroscopy (XPS).

The electromagnetic (EM) parameters of the sample were tested using a Vector Network Analyzer (Agilent N5244A) over a frequency range of 2.0–18.0 GHz. Before the test, the sample and paraffin were weighed according to the specific proportion, melted at 60 °C, fully mixed, and then transferred to a mold with a radius of 7.0 mm, an inner diameter of 3.04 mm, and a thickness of 2.0 mm. The mixture was then pressed into a concentric ring.

## 3. Results and discussion

### 3.1 Design of mPM

**Fig. 1:**
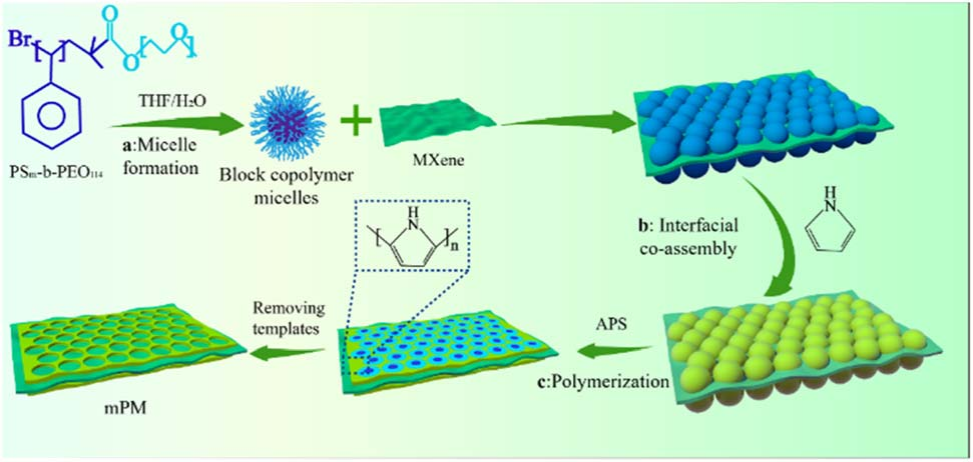
Schematic diagram of mPM.

In this work, we demonstrate an approach for prepare rational growth of a 2D mesoporous PPy network on MXene, yielding 2D sandwich-like mesoporous polypyrrole/MXene (mPM) composite, as shown in Fig. 1. The Process (a): a certain amount of PS_*m*_-*b*-PEO_114_ block copolymer (the subscript *m* express the degree of polymerization) was dissolved in THF/H_2_O solvent mixture to form spherical micelles. Process (b): subsequently, the DL-MXene and pyrrole (Py) monomer were introduced in micellar solution. The interfacial self-assembly of the micelles and Py on MXene sheets is driven by hydrogen bonding and electrostatic interactions. Process (c): the polymerization was initiated by adding APS solution and the PS_*m*_-*b*-PEO_114_ was removed by washing the product with THF, EtOH, and H_2_O to obtain mPM composite.

### 3.2 Morphology and structure

**Fig. 2:**
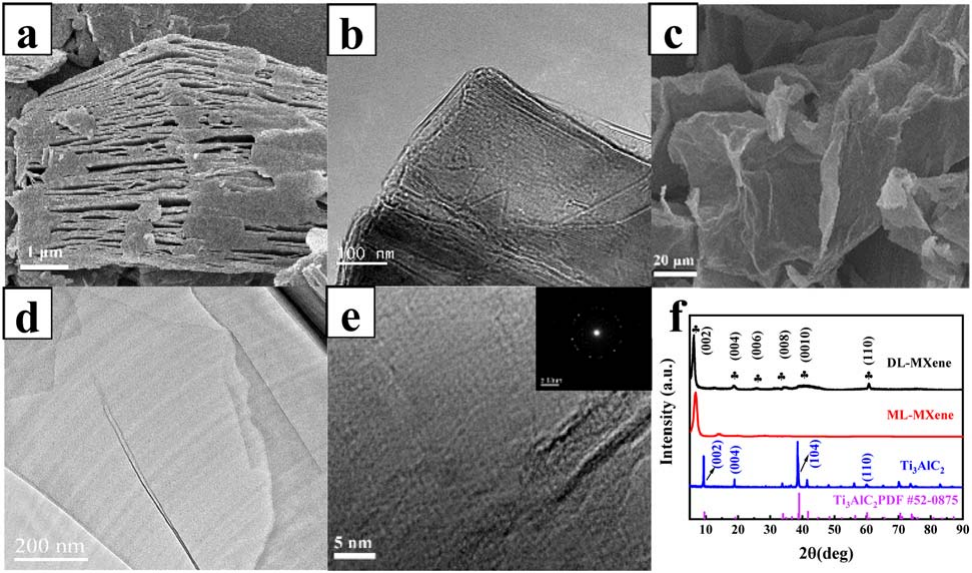
SEM images of ML-MXene (a) and DL-MXene (c). TEM images of ML-MXene (b) and DL-MXene (d). HRTEM (e) and SAED patterns (inset) of DL-MXene. XRD patterns of DL-MXene, ML-MXene, and Ti_3_AlC_2_ (f).

The Al layer in the Ti_3_AlC_2_ (MAX) phase was etched to obtain the ML-MXene by *in situ* hydrofluoric acid method, and then was exfoliated by ultrasound-assisted method to obtain DL-MXene. The structure and morphologies of the MXene were first analyzed by SEM, TEM, and XRD as presented in Fig. 2. As shown in Fig. 2a, the removal of the Al phase imparts an accordion-like layered structure to the ML-MXene, which exhibits a typical 2D laminar structure with lamellae that have a transverse size exceeding 3 μm. The cross-sectional structure of ML-MXene, analyzed by TEM (Fig. 2b), reveals that the nanosheets are neatly stacked, forming a multilayer structure with highly ordered sub-nanometer interlayer channels. The ML-MXene was subjected to assisted exfoliation treatment using high-power ultrasound to obtain DL-MXene nanosheets with larger sizes. From the SEM image of DL-MXene in Fig. 2c, the prepared nanosheets exhibited a wrinkled and curled nanosheet shape, and the TEM image (Fig. 2d) showed that DL-MXene appears as an almost transparent thin sheet with a slight fold, which indicated that it had been exfoliated into a monolithic sheet. The surface shows no apparent pinholes, cracks, or breakage, suggesting that the integrity of the sheet is well preserved, and there are almost no nanoscale defects. The transverse size of the nanosheets is approximately 600 nm. According to the HRTEM photographs and Selected Area Electron Diffraction (SAED) spectra (Fig. 2e), the DL-MXene nanosheets showed a basal hexagonal crystal system structure, and the sheet had a better degree of crystallization.

ML-MXene was synthesized by selectively etching the Al layer from the MAX phase Ti_3_AlC_2_ using the LiF–HCl system. XRD analysis (Fig. 2f) was employed to confirm the successful etching and delamination of Ti_3_C_2_.^36^ The XRD spectrum of MAX in Fig. S1† displays two distinct characteristic peaks: the (104) peak at 2*θ* = 39°, indicating the Al atomic layer, and the (002) peak at approximately 2*θ* = 9.5°, reflecting the interlayer spacing, both of which match well with the card of the Ti_3_AlC_2_ MAX phase material (PDF #52-0875). As the Al layer is etched, the original crystal structure is disrupted, the X-ray absorption is reduced, and the disappearance of the strongest peak (104) confirms the successful etching of the MAX phase and the transformation of Ti_3_AlC_2_ into Ti_3_C_2_T_*x*_ MXene. Furthermore, the (002) peak of the MXene shifts to a lower Bragg angle compared to the MAX phase, due to the intercalation of water and ions, as well as the expansion of the interlayer spacing caused by the functional groups (–O, –OH, –F, and –Cl) replacing the Al atomic layer. The (002) peak of DL-MXene is slightly displaced to the left relative to ML-MXene, likely due to the delamination process induced by ice-bath sonication. In contrast, the (002) peak of ML-MXene is shifted to approximately 7.82°, which corresponds to the lattice parameter (*c*-LP) = 22.6 Å.^37^ Moreover, the appearance of the standard diffraction peak crystal planes (002), (004), (006), (008), (0100), and (110) of MXene further indicates that MXene nanosheets were obtained.

**Fig. 3:**
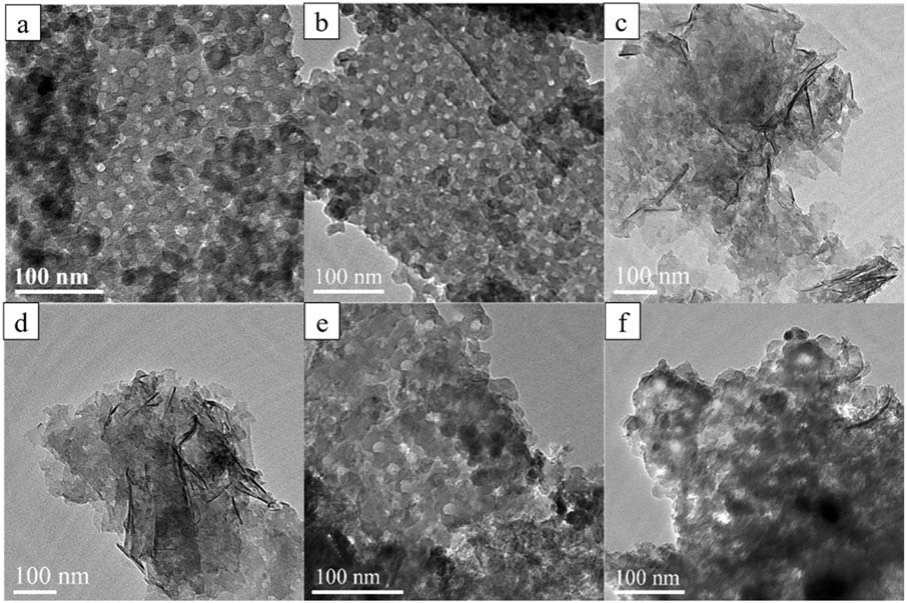
TEM images of mPM with different MXene and Py mass ratios: mPM-0.5 (a), mPM-1 (b), mPM-1.5 (c), and mPM-2 (d), and mPM with different pore sizes (MXene : Py = 1): mPM-50 (e), mPM-150 (f).

The influences of the MXene/Py ratio and pore size on the morphologies of mPM composite were systematically investigated. The TEM images provide a clear representation of the micromorphology of mPM with varying PPy ratios, as shown in Fig. 3a–d. The mPM exhibits a layered structure with tightly packed spherical pores and a uniform pore size. On the one hand, the mPM can be observed as a sandwich structure from the edges of the lamellae, with PPy polymerized on both sides of DL-MXene. On the other hand, the pore sizes of the TEM images were counted (Fig. S1†), and the pore size was about 18 nm, which was very close to the diameter of the micelle, proving that the PS_100_-*b*-PEO_114_ micelle template effectively induced the formation of the PPy mesoporous structure.^38^ Furthermore, PPy also forms a conductive network, which increases the conductive pathway of mPM. As the MXene content increases, PPy gradually fails to fully cover the surface of the MXene; however, the presence of pores remains observable. Remarkably, when EMWs are incident into the absorbing composite, certain defects and apertures in the composite can cause dipole polarization and increase the dielectric loss, thus promoting the improvement of the microwave absorption performance of the composite. Fig. 3b, e, and f show the TEM images of three mPM samples with different pore sizes, prepared with a MXene-to-Py mass ratio of 1. The samples, mPM-50, mPM-1, and mPM-150, were synthesized using PS_50_-*b*-PEO_114_, PS_100_-*b*-PEO_114_, and PS_150_-*b*-PEO_114_ templating agents, respectively. The pore size analysis of the three TEM images shows that the pore size of mPM-50 is about 15 nm, mPM-1 is about 18 nm, and mPM-150 is about 24 nm, all of which are very close to the diameter of the micelles.

**Fig. 4:**
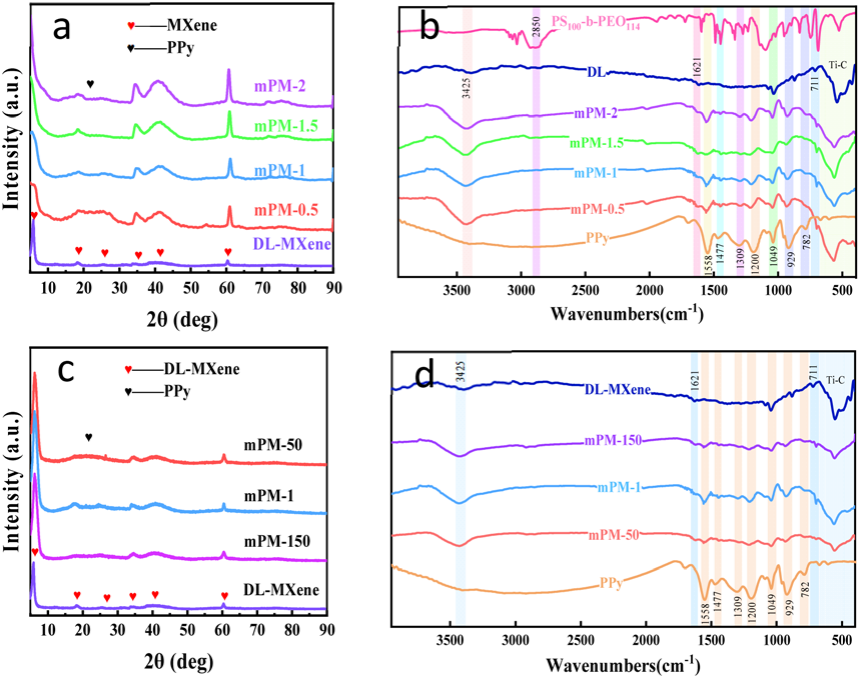
XRD pattern (a), FT-IR spectra (b) of mPM with different MXene ratios; XRD pattern (c), FT-IR spectra (d) of mPM with different pore sizes (MXene : Py = 1).

XRD, FT-IR, and XPS analyses were performed to confirm the synthesis of mPM. Fig. 4a shows the XRD patterns of mPM with different MXene ratios. It is evident that the weak absorption peaks of PPy at 15–30° made up the peaks of mPM and the MXene peaks of (002), (004), (006), (0010) and (110) crystal plane compositions, the intensity of (002) peak with the increase of MXene content, and the amorphous carbon peak of PPy gradually weakens. In addition, the layer spacing of DL-MXene was calculated to be 14.43 Å based on the characteristic peak of (002) located at 6.12°, whereas mPM-1 (002) shifted to 5.54° and the crystalline facet spacing increased to 15.94 Å. This significant change was attributed to the enhancement of MXene layer spacing as a result of the insertion of various PPy contents.^39^

Fig. 4b shows the FT-IR spectra of DL-MXene, PPy, mPM, and PS_100_-*b*-PEO_114_. In the spectra of DL-MXene,^40^ the typical absorption peaks are the Ti–C stretching vibration peak at 565 cm^−1^ and the stretching vibration peaks of –OH at 3425 cm^−1^ and 1621 cm^−1^. In the spectra of pure PPy, the peaks of 1558 cm^−1^ and 1447 cm^−1^ belong to the vibrational stretching of the Py ring,^41^ while the N–H in-plane vibration at 1049 cm^−1^ and the C=N ring deformation at 1200 cm^−1^ and the C–H in-plane deformation at 929 cm^−1^ are characteristic peaks of PPy. In the spectra of the mPM composites, the Ti–C stretching vibration peak at 565 cm^−1^ is observed, as well as the characteristic peaks of PPy in all mPM composites. Additionally, the PPy peaks in the mPM composites at 1558 cm^−1^, 1477 cm^−1^, 1309 cm^−1^, 1200 cm^−1^, 1049 cm^−1^, 929 cm^−1^, and 782 cm^−1^ exhibit certain shifts.^42^ These shifts may result from the π–π interaction between MXene and PPy, which decreases the electron cloud density on the conjugated π-bonds and alters the energy of the conjugate bonds, leading to the shifting of the characteristic peaks of doped PPy.^43^ This phenomenon indicates the successful integration of PPy and MXene. Finally, we find that the characteristic peak of the stretching vibration of –CH_2_ possessed by the polymer template PS_100_-*b*-PEO_114_ at 2850 cm^−1^ completely disappears, indicating that the polymer template has been completely washed away by THF.

Fig. 4c shows the XRD spectra of mPM with different pore sizes when the MXene : Py ratio is 1. The XRD patterns show a broad amorphous peak at about 25° that corresponds to amorphous PPy.^44^ The (002) and (110) crystal planes of MXene were represented by the characteristic peaks at 5.7° and 60.6°, respectively. This suggests a well-mixed state of PPy and MXene within the composites. Fig. 4d displays the FT-IR spectra of mPM with different pore sizes, revealing that the peaks for mPM-50, mPM-1, and mPM-150 correspond to those of PPy and DL-MXene. Notably, the PPy peaks in the mPM composites display similar shifts due to π–π interactions, further confirming the successful incorporation of PPy with DL-MXene.

The C 1s spectrum of mPM-1 composite is presented in Fig. S2a.† The peaks observed at binding energies of 283.61 eV, 284.80 eV, 286.48 eV, 288.01 eV, and 290.92 eV are assigned to Ti–C, C–C/C=C, C–O, C=O, and C–F bonds, respectively. Notably, the peak at 285.64 eV, corresponding to the C–N bond, likely originates from the interfacial interaction between MXene and PPy, suggesting covalent bonding at the interface. Furthermore, Fig. S2b† displays the N 1s spectrum of the mPM-1 composite, where the peaks at 399.93 eV and 402.71 eV are attributed to C–N and –NH_2_ bonds, respectively. These findings provide strong evidence that the surface functional groups of MXene are covalently bonded to the molecular chains of PPy, confirming the formation of a robust interfacial connection between the two components.

### 3.3 EM parameters of absorbing materials

EM parameters (the complex permittivity and complex permeability) are widely recognized as key factors in determining microwave absorption performance. The complex permittivity and the permeability have both imaginary (*ε*′′ and *μ*′′) and real components (*ε*′ and *μ*′). The imaginary part represents the loss capacity of EM energy, whereas the real part reflects the storage capacity of EM energy. The following equations illustrate the correlation between *ε*′ and imaginary *ε*′′ values using the Debye theory:1
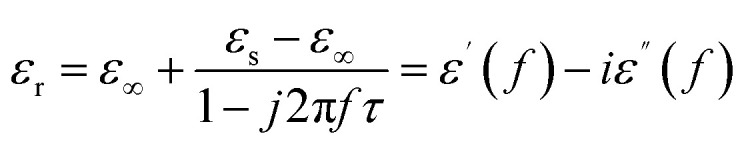
2
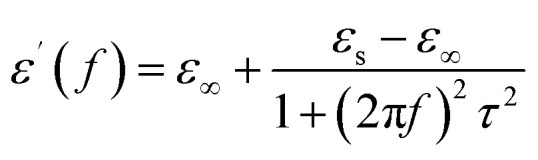
3
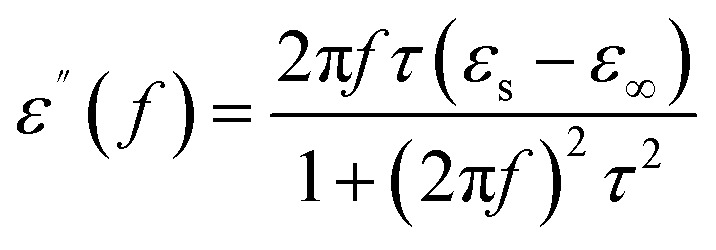
where *j* is the imaginary unit, *f* is the EMWs frequency, *τ* is the relaxation time, *ε*_s_ is the static permittivity, and *ε*_∞_ is the relative permittivity at the high-frequency limit. From eqn (1)–(3), there is a polarization relaxation behavior in these composites during the absorption of EMWs in terms of Debye's theory.

**Fig. 5:**
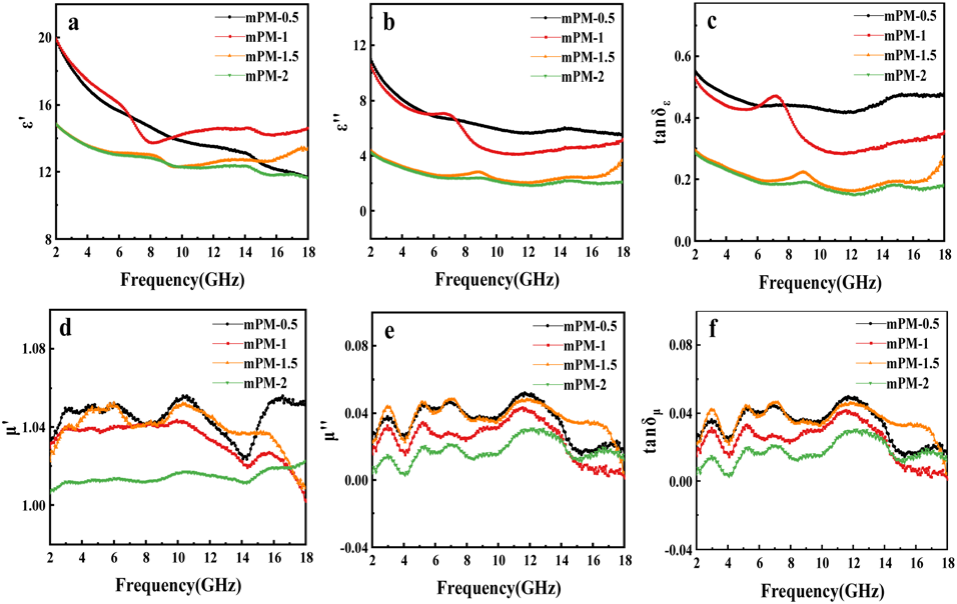
The frequency dependence of the real (a) and imaginary (b) parts of the complex permittivity, the dielectric loss tangent (c), the real (d) and imaginary (e) parts of the complex permeability, the magnetic loss tangent (f) for mPM (different MXene/Py ratios and the same template PS_100_-*b*-PEO_114_, paraffin ratio 30 wt%).

Fig. 5a–f shows the EM parameter distribution of mPM with different MXene/Py ratios in the frequency range of 2 to 18 GHz when the paraffin ratio is 30 wt%. As shown in Fig. 5a and b, the *ε*′ values of mPM-0.5, mPM-1, mPM-1.5, and mPM-2 vary in the ranges of 19.93–11.64, 19.94–13.75, 14.85–12.26 and 14.85–11.62, respectively, and revealed a downward trend, which was brought on by the enhanced interface polarization between MXene and PPy as well as the periodic change of the orientation polarization lag electric field of the electric dipole. The *ε*′′ values of mPM-0.5, mPM-1, mPM-1.5, and mPM-2 vary in the ranges of 10.99–5.54, 10.57–4.12, 4.39–2.02, and 4.25–1.84, respectively. The *ε*′′ value of mPM-0.5 and mPM-1 are higher than those of mPM-1.5 and mPM-2, with a progressive increase in *ε*′′ as the PPy content rises. This is because the conductive network of mPM becomes more complete with higher PPy content, thereby enhancing conductivity. Therefore, the dielectric loss of mPM-0.5 and mPM-1 is the strongest. Additionally, multiple resonance peaks are observed in the *ε*′′ spectra of all mPM composites, indicating the presence of multiple polarization relaxation processes.

In conclusion, both the real part (*ε*′) and imaginary part (*ε*′′) of the permittivity of all mPM composites decrease with increasing frequency, indicating that all mPM composites exhibit varying degrees of frequency dispersion, which facilitates the dissipation of EMWs.^45^ The findings demonstrated that *ε*′ and *ε*′′ show an increasing tendency as the PPy content increases, suggesting that PPy improved the dielectric properties of mPM. The tan *δ*_*ε*_ value and tan *δ*_*μ*_ value represent the dielectric loss capacity and magnetic loss capacity of the mPM respectively. As can be seen from Fig. 5c and f, the dielectric loss capacity of the mPM generally shows a decreasing trend with the increase of frequency and the tan *δ*_*ε*_ curve shows two obvious peaks, indicating the occurrence of double dielectric relaxation during the EMW loss process.^46^ In Fig. 5d and e, the real part (*μ*′) and imaginary part (*μ*′′) of complex permeability are about 1.0 and 0.0, respectively, indicating the absence of a magnetic component in the composite. As a result, the composite can be regarded as a non-magnetic material. The tan *δ*_*μ*_ values of the four different mPMs are all around 0.0, indicating that the magnetic loss can be ignored in the non-uniform system. In the whole measurement frequency range, the dielectric loss capacity of mPM is extremely stronger than the magnetic loss capacity, demonstrating that the loss mechanism of all mPM to EMWs is mainly dielectric loss.

**Fig. 6:**
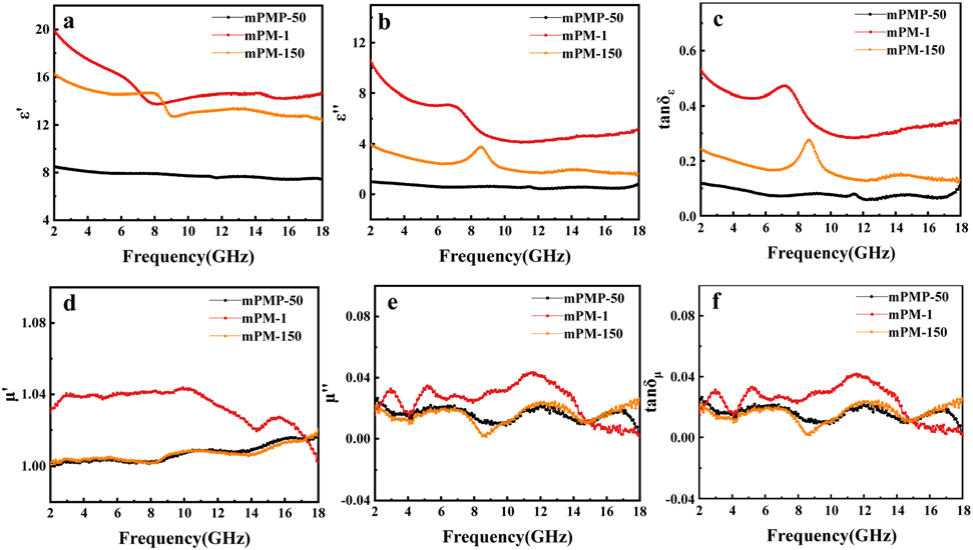
The frequency dependence of the real (a) and imaginary (b) parts of the complex permittivity, the dielectric loss tangent (c), the real (d) and imaginary (e) parts of the complex permeability, the magnetic loss tangent (f) for mPM (different PS_*m*_-*b*-PEO_114_ templates, the same MXene/Py ratios (1 : 1), paraffin ratio 30 wt%).

Fig. 6 shows the EM parameter distributions of mPM with different pore sizes in the frequency range of 2–18 GHz when the MXene : Py ratio is 1 and the paraffin ratio is 30 wt%. As shown in Fig. 6a and b, the permittivity of three mPMs as frequency increases, demonstrating a remarkable frequency dispersion phenomenon. In the range of 2–18 GHz, the *ε*′′ values of mPM-1 are higher than those of mPM-50 and mPM-150, indicating that the mPM-1 possesses the highest dielectric loss. As shown in Fig. 6c, the tan *δ*_ε_ curves for mPM with different pore sizes display two distinct peaks, further suggesting the occurrence of double dielectric relaxation during the EMWs loss process. In addition, since the composite does not contain magnetic components, the real (*μ*′) and imaginary (*μ*′′) parts of the complex permeability in Fig. 6d and e are about 1.0 and 0.0, respectively, and the magnetic loss is negligible. The primary cause of the EMWs loss mechanism of mPM is dielectric loss since tan *δ*_*ε*_ is much higher than tan *δ*_*μ*_. In summary, the mPM-1 prepared using polymer PS_100_-*b*-PEO_114_ has a stronger dielectric loss, and mPM-1 with 18 nm pore sizes is more favorable for EMWs absorption.

### 3.4 Microwave absorption properties

The following formulas are used to calculate the RL of materials in light of the transmission line theory to assess their performance in terms of microwave absorption.^47,48^4
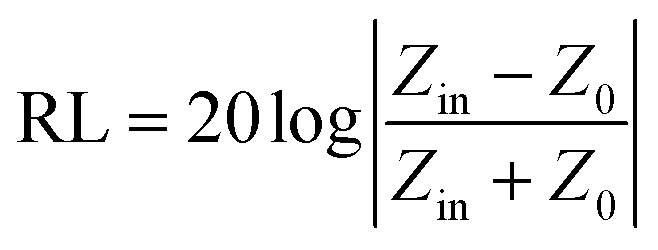
5
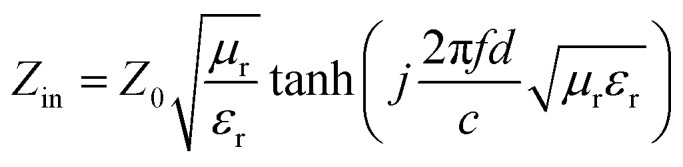
The frequency, speed of light, thickness, complex permeability, and complex permittivity are denoted by *f*, *c*, *d*, *μ*_r_, and *ε*_r_, respectively. The input impedance is *Z*_in_, while the free space impedance is *Z*_0_.

To evaluate the microwave absorption performance of the composite, the RL of the material under the thickness of 1.0–5.0 mm is simulated by eqn (4) and (5) based on the measured permittivity and permeability under the transmission line theory in the frequency band of 2–18 GHz. In general, the frequency range where RL < −10 dB is the EBW of composite since it can efficiently absorb over 90% of the incident EMWs when the RL value is less than −10 dB.

**Fig. 7:**
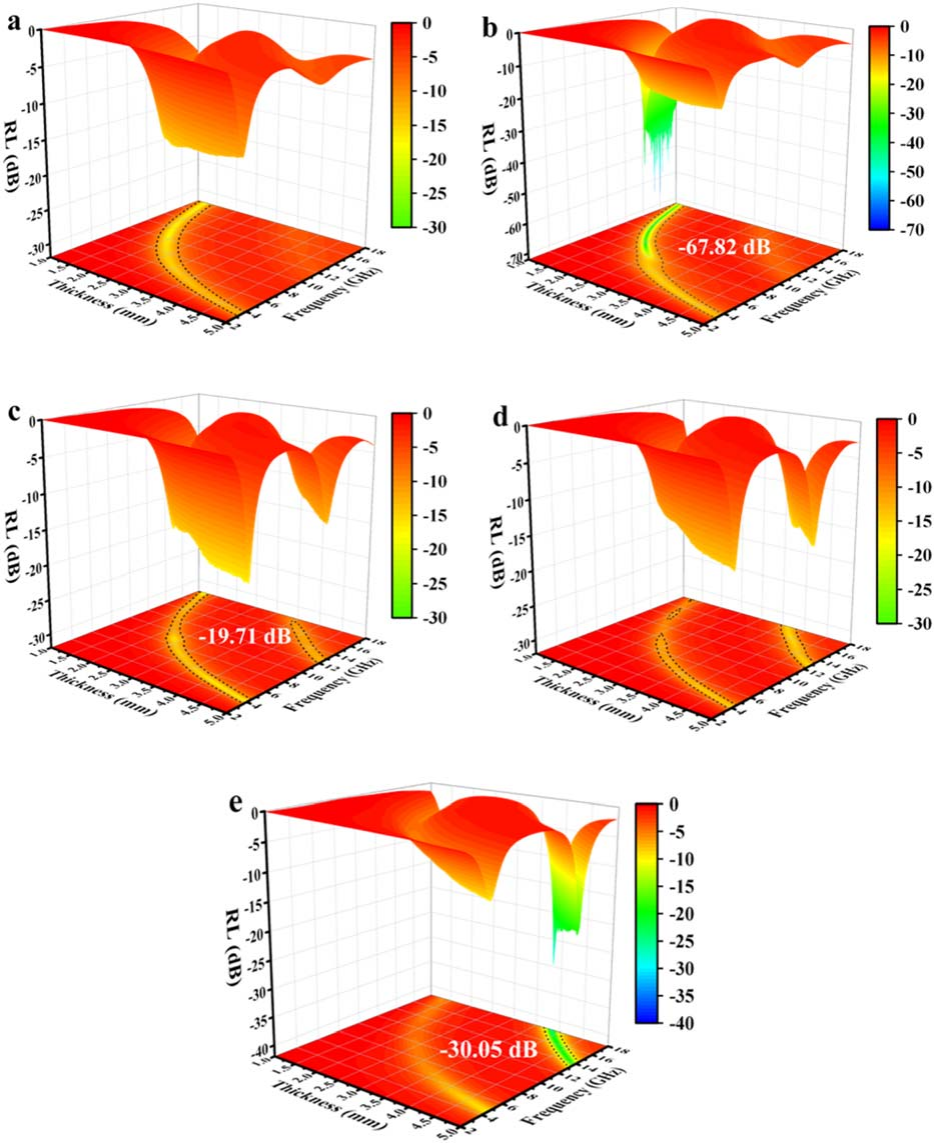
The 3D reflection loss patterns of mPM: mPM-0.5 (a), mPM-1 (b), mPM-1.5 (c), and mPM-2 (d) and the 3D reflection loss patterns of mesoporous PPy/MXene/Fe_3_O_4_ (e).

When a material absorbs more than 90% of the incident EMWs (RL ≤ −10 dB), it is generally considered an excellent microwave absorber. To elucidate the EMWs absorption performance of the obtained mPM composites, three-dimensional graphs of RL at a given frequency and layer thickness were calculated and plotted using the measured EM parameters. As shown in Fig. 7, the RL values for all mPM composites are below −10 dB, indicating that all the composites exhibit EMW loss capabilities. Specifically, the mPM-0.5 with a thickness of 1.2 mm achieves the optimal RL of −17.54 dB at 17.92 GHz, and the maximum EBW is 4.72 GHz (13.28–18 GHz) (Fig. 7a). As shown in Fig. 7b, when the ratio of MXene to Py is adjusted to 1, the RL decreases significantly and the EBW increases, thus improving the EMWs absorption performance. The RL of mPM-1 reaches −67.82 dB at a thickness of 1.37 mm and a frequency of 14.4 GHz, with an EBW of 3.44 GHz (12.88–16.32 GHz). mPM-1 possesses the maximum EBW of 3.68 GHz (14.16–17.84 GHz) when the thickness is 1.25 mm and the frequency is 15.92 GHz (Table S1†). As shown in Fig. 7c, mPM-1.5 achieves a minimum RL of −19.70 dB at 1.16 mm and 18.0 GHz, with an EBW of 1.12 GHz (16.88–18 GHz). When the ratio of MXene to Py is further increased to 2, as shown in Fig. 7d, mPM-2 has the minimum RL value of −15.51 dB at a thickness of 3.95 mm and a frequency of 16.4 GHz, with an EBW of 1.44 GHz (15.75–17.2 GHz).

In addition, we also studied the effect of Fe_3_O_4_ loading on the microwave absorption performance of porous mPM composites, specifically the mesoporous PPy/MXene/Fe_3_O_4_ (mPMF) composites (Fig. 7e). When the ratio of MXene to Fe_3_O_4_ is 1, an RL value of −30.05 dB is obtained at 3.83 mm. However, the loading of Fe_3_O_4_ caused pore blockage, which hindered electron transport and reduced dielectric loss, resulting in the mPMF composites failing to achieve the desired microwave absorption performance. A combination of TEM and EM parameter characterization shows that the interface between mesoporous PPy and MXene has the largest polarization and the strongest polarization loss. The results show that better microwave absorption performance is achieved by constructing mPM with a lamellar sandwich structure. The introduction of mesopores further enhances the EMWs absorption properties of the composite, the matching thickness decreases, and the EBW increases.

**Fig. 8:**
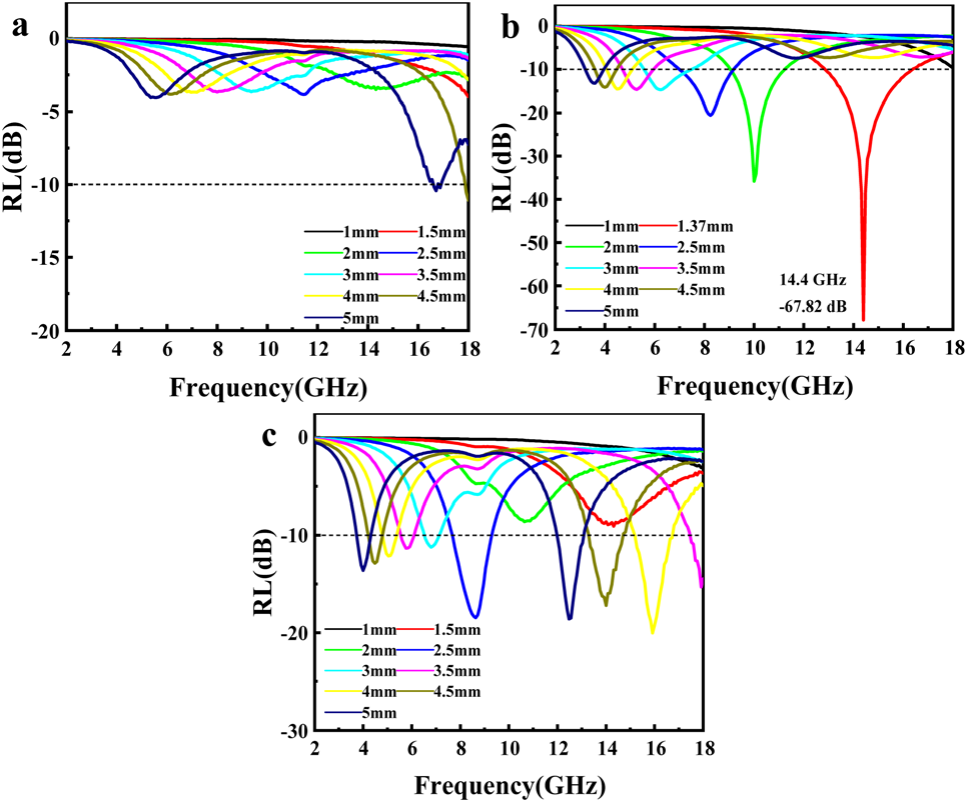
RL of mPM with different pore sizes (MXene : Py = 1): mPM-50 (a), mPM-1 (b) and mPM-150 (c).

The RL of mPM with different pore sizes at a given frequency and different layer thicknesses can be computed using the obtained EM parameters. As shown in Fig. 8a, the minimum RL of mPM-50 is −11.08 dB, which weakens the capacity of EMWs loss. In contrast, mPM-1 shows a high EMWs absorption capability as shown in Fig. 8b. Its minimum RL value reaches −67.82 dB at a thickness of 1.37 mm and a frequency of 14.4 GHz, with an EBW of 3.44 GHz (12.88–16.32 GHz). When the pore size is further enlarged, the RL of mPM-150 decreases, as shown in Fig. 8c, where the mPM-150 has a minimum RL of −20.03 dB at 15.92 GHz with a thickness of 4.0 mm, with an EBW of 1.58 GHz (15.12–16.70 GHz). Thus, it can be found that the mPM-1 prepared using the polymer PS_100_-*b*-PEO_114_ has the thinnest matched thickness, the highest EMWs absorption capacity, and the widest EBW (Table S2†).

### 3.5 The proposed mechanism for microwave absorption of mPM

To gain further insights into the microwave absorption mechanism of mPM, the dielectric relaxation process was investigated by Debye's theory. The eqn (6) of the Cole–Cole semicircle was obtained from eqn (2) and (3). Each semicircle represents a single Debye relaxation process, and the *ε*′–*ε*′′ curves can be used to reflect the shape of the Cole–Cole semicircles.6
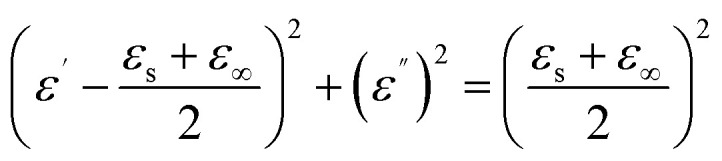


**Fig. 9:**
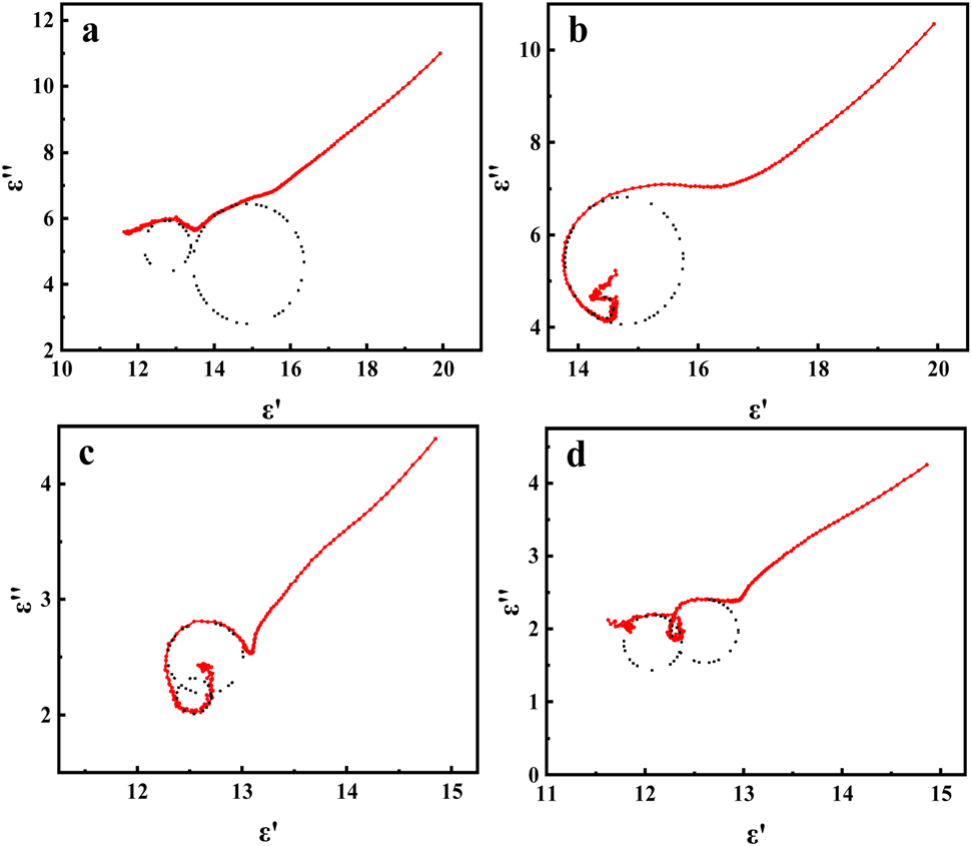
Cole–Cole curves of mPM: mPM-0.5 (a), mPM-1 (b), mPM-1.5 (c), and mPM-2 (d).

Fig. 9 shows the Cole–Cole plots of all mPM composites, which exhibit two clear semicircles with tails that are upwardly and obliquely curved. According to the modified Debye relaxation theory, this again indicates the existence of two relaxation polarizations. This is because the porous sandwich structure will contain more heterogeneous interfaces to enhance the polarization loss due to interfacial polarization. At the same time, the increase of unsaturated dangling bonds on the MXene surface will form more polarization centers, thus enhancing the polarization loss caused by dipole polarization. Therefore, two Cole–Cole circles can be clearly seen in Fig. 9. The upward-curved linear tail also suggests the presence of significant conductive losses. From the above discussion, it can be determined that the microwave attenuation capability of the studied mPM mainly originates from dielectric loss.

Generally, the value of *Z* = |*Z*_in_/*Z*_0_| (eqn (7)) is used to assess impedance matching, which plays a crucial role in determining the microwave absorption performance of absorbing materials,^49^ as shown in eqn (7):7
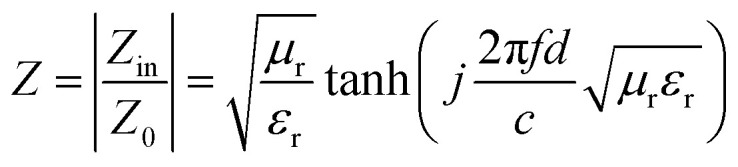
In this equation, *d* represents the sample thickness, *f* stands for EMWs frequency, *c* is the speed of EMWs, *Z*_0_ is the free space impedance, and *Z*_in_ is the input impedance. When the value of *Z* is 1, the material exhibits optimal impedance matching, allowing the maximum amount of EMWs to penetrate the material.

Attenuation characteristics are another important factor in determining the microwave absorption performance of absorbing materials. Specifically, for EMWs entering the interior of the absorbing material, various loss mechanisms convert and dissipate the energy within the material, thereby minimizing the reflection of EMWs energy back to the exterior of the material.^50^ The attenuation characteristics of absorbing materials are shown in eqn (8):8
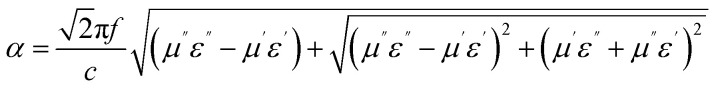
where *f* is the frequency and *c* is the light velocity.

In eqn (8), *α* represents the attenuation constant of the absorbing material, and a large attenuation constant implies strong attenuation characteristics. It can be observed that the imaginary parts of the complex permeability and complex permittivity are most likely to contribute to a stronger attenuation constant for the absorbing material. However, an excessive imaginary component of the complex permittivity may negatively impact impedance matching. Therefore, to guarantee good impedance matching and good attenuation characteristics, the balance of the EM parameters must be adjusted during the design and synthesis of the microwave absorbing materials. To analyze the EMWs absorption mechanism of mPM, it is well known that the corresponding microwave absorption theory can also use impedance matching to explain the variations in microwave absorption performance.

The impedance matching value of the sample is calculated by eqn (5) to ascertain the extent of the incident EMWs entered the absorber, which is characterized by drawing a plot of impedance matching *versus* frequency. When the value of impedance matching approaches 1, it shows that the material can completely absorb EMWs, which is equivalent to having good impedance matching characteristics.

**Fig. 10:**
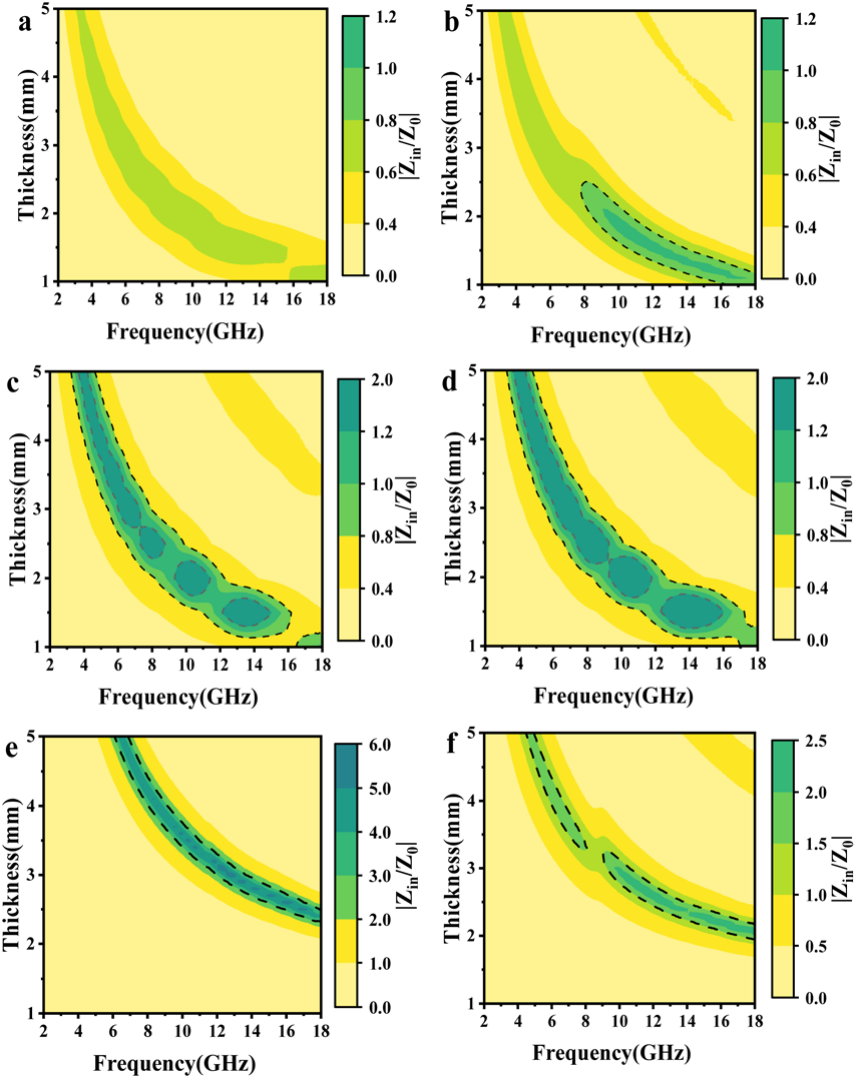
Color plots of *Z*-values of mPM from 1.0 to 5.0 mm thickness at 2.0–18.0 GHz: mPM-0.5 (a), mPM-1 (b), mPM-1.5 (c), mPM-2 (d), mPM-50 (e), and mPM-150 (f).

The color plots of the *Z*-values for different mPM composites with thicknesses ranging from 1.0 to 5.0 mm over the frequency range of 2.0 to 18.0 GHz are presented. From Fig. 10a, c, and d, it can be found that when the proportion of Py is too high, the impedance matching values of mPM-0.5 are all less than 0.8, resulting in EMWs reflection. Conversely, when the proportion of Py is too low, mPM-1.5 and mPM-2 exhibit excessive dielectric loss, leading to impedance mismatch. As seen in Fig. 10b, mPM-1 demonstrates good impedance matching over a wide frequency range when the thickness is 1.25 mm. Under these conditions, most of the EMWs can penetrate the composite material and be converted into thermal energy through the dielectric loss mechanism, achieving effective attenuation within the material.

A suitable pore size can significantly enhance the impedance matching of the absorbing material. The small pore size of mPM-50 (Fig. 10e) may hinder the incidence of EMWs, preventing effective internal attenuation and resulting in impedance mismatch. In addition, the mPM-150 composite also (Fig. 10f) has an excessive impedance match, but it is less than that of mPM-50, which is more favorable for the incidence of EMWs. Therefore, excessively large pores may also lead to the reflection of part of the EMWs, reducing the amount of the structure attenuation of EMWs. In summary, the mPM-1 composite with a pore size of 18 nm demonstrates the best impedance matching.

**Fig. 11:**
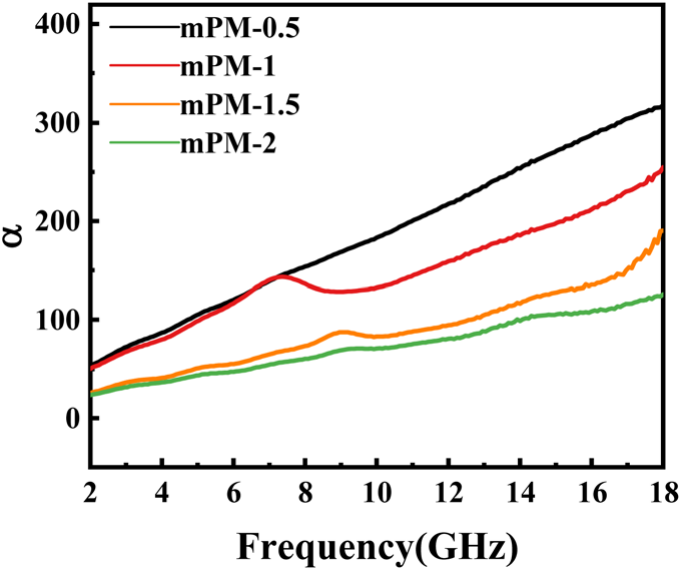
The plot of attenuation constant *α versus* frequency for mPM.

As shown in Fig. 11, the attenuation constants *α* of mPM-0.5 and mPM-1 are almost always higher than those of mPM-1.5 and mPM-2 in the whole frequency range. Herein, the differences in the *α* curves of mPM-0.5 and mPM-1 are not obvious. Thus, mPM-0.5 and mPM-1 exhibit excellent microwave absorption performance. The enhanced EMWs attenuation capability is derived from the improved synergistic effect of dipole polarization and interfacial polarization.

**Fig. 12:**
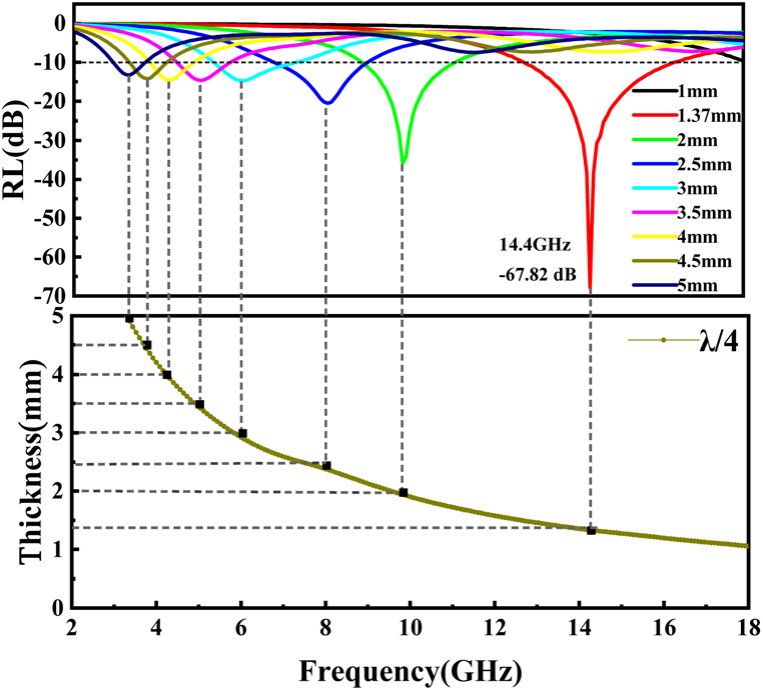
*λ*/4 wavelength matching model of mPM-1.

To achieve the interference loss, the thickness of the material should conform to the *λ*/4 model,^51^ indicating that EMWs will interfere with each other and be eliminated in the absorbers, as shown in the following:9
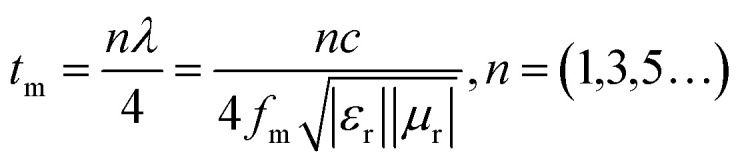
The uneven number, light velocity, EMWs wavelength, peak frequency, and matching thickness are represented by *n*, *c*, *λ*, *f*_m_, and *t*_m_, respectively. When *n* is an odd number, there is a difference of *λ*/2 between the reflected wave 1 and the reflected wave 2. At this time, the phases of the two reflected waves are opposite and interfere with each other to offset the energy of the two EMWs and achieve the purpose of consuming the energy of the EMWs. In addition, it is equally obvious from the RL curves that the minimum RL values of all samples gradually shift to the lower frequencies with the increase of thickness, which is attributed to the existence of the *λ*/4 attenuation phenomenon. Fig. 12 shows the *λ*/4 model of mPM-1. It is evident that, as the layer thickness increases, the frequency corresponding to the minimum RL value of the RL curve decreases.

To further characterize the relationship between the reflection loss of the material, the frequency of the incident EMWs, and the thickness of the sample, the EM parameters of mPM-1 were substituted into the equation shown in eqn (9). Since only one loss peak occurs for each thickness of the composite, *n* = 1 was used during the calculation process, and the *λ*/4 model curve was then calculated and compared with the RL curve of the mPM-1. The frequency of the absorption peaks at each thickness and the trend of the matching thickness are in accordance with the obtained theoretical values calculated by eqn (9), indicating that the loss mechanism of mPM-1 is in accordance with the *λ*/4 model. Therefore, when the EMWs enter the inside of the composite, part of the EMWs reflected by the substrate interferes with the incoming EMWs and cancels the phase, which is an important factor for the EMWs to be absorbed by the composite.

**Fig. 13:**
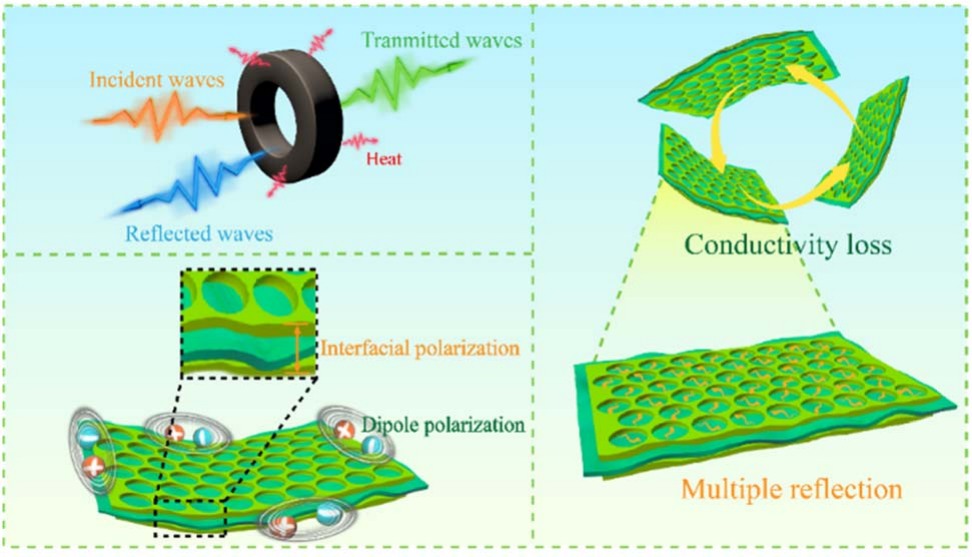
Mechanism of wave absorption in mPM.

Based on the results presented above, it can be concluded that the wave absorption mechanism of the mPM with lamellar sandwich structure is shown in Fig. 13: (i) the appropriate ratio of assembling PPy can improve the conductivity of the composites, thereby enhancing the dielectric loss. (ii) The porous PPy layer in the lamellar sandwich structure forms numerous interfaces, such as PPy–MXene, PPy–air, and MXene–air, which intensify the interfacial polarization of the composite and contribute to an increase in dielectric loss. (iii) The porous structure promotes the dissipation of electromagnetic wave energy, providing more space for scattering, which extends the propagation path and facilitates the attenuation of electromagnetic waves. (iv) The increase in unsaturated bonds on the surface of the porous structure creates more polarization centers, enhancing dipole steering polarization and further increasing dielectric loss. (v) MXene and PPy form a well-developed conductive network in the matrix, which promotes electron hopping and migration and improves microwave absorption energy consumption.

## 4. Conclusions

In this paper, mPM composite with a sandwich structure was synthesized by an assembling PPy on both sides of MXene. The molecular synergistic self-assembly strategy was used to regulate the mesoporous size of the mPM composite with the polymerization of the template agent. The results show that appropriate mesoporous structure and heterogeneous interfaces can regulate the impedance matching and enhance dielectric loss of the composites, thus improving the EMWs absorption performance. When the ratio of MXene to Py is 1, mPM-1 exhibits excellent EMWs absorption performance with an RL value of −67.82 dB at 14.4 GHz. Furthermore, the impedance matching of the composite can be significantly improved with appropriate pore size, primarily due to the multiple reflections at the heterogeneous interfaces and the synergistic effects between the layered MXene and the conductive PPy network, which effectively enhance the electromagnetic wave absorption performance. Moreover, the different heterogeneous interfaces enhance the interfacial polarization effect of the composite, while the abundant functional groups of MXene and the increase in unsaturated bonds on the surface provide more polarization centers, strengthening dipole orientation polarization and further enhancing dielectric loss. This study provides guidance for the design of superior MXene composite structures with dielectric loss-type microwave absorbing performance.

## Data availability

The datasets generated and/or analyzed during the current study are available from the corresponding author on reasonable request.

## Author contributions

Wenjuan Zhang: conceptualization, methodology, writing – review and editing, and supervision. Xiangyue Yang: writing – original draft and formal analysis. Youliang Wang: writing – review and editing. Yaxian Wang: supervision. Xuyang Wu: validation and data curation. Yongqian Shen: funding acquisition. All authors have accepted responsibility for the entire content of this manuscript and approved its submission.

## Conflicts of interest

The authors declare that they have no known competing financial interests or personal relationships that could have appeared to influence the work reported in this paper.

## Supplementary Material

RA-015-D5RA00972C-s001
