# Supplementary material for: Fabrication of a tunable mesoporous polypyrrole/MXene composite with a sandwich structure for enhancing electromagnetic wave absorption performance
Source: RSC Adv. 2025 Apr 3;15(13):10298–309. doi: 10.1039/d5ra00972c (PMC11966190; doi:10.1039/d5ra00972c)
Supplement: RA-015-D5RA00972C-s001 [file RA-015-D5RA00972C-s001.pdf]

Supplementary Information (SI) for RSC Advances.
This journal is © The Royal Society of Chemistry 2025

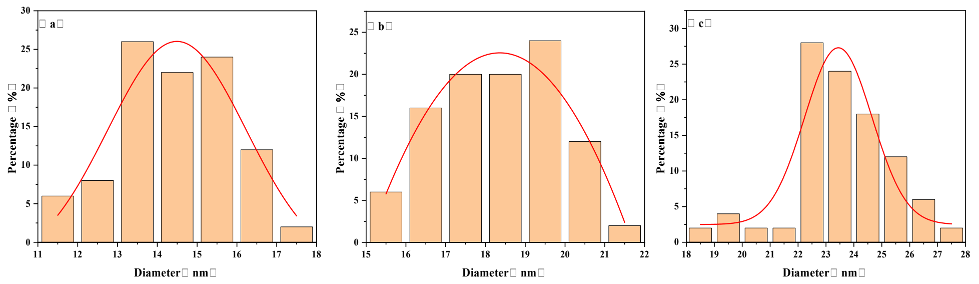

Fig. S1 The average pore size distribution of mPM-50 (a), mPM-1 (b), and mPM-150 (c).

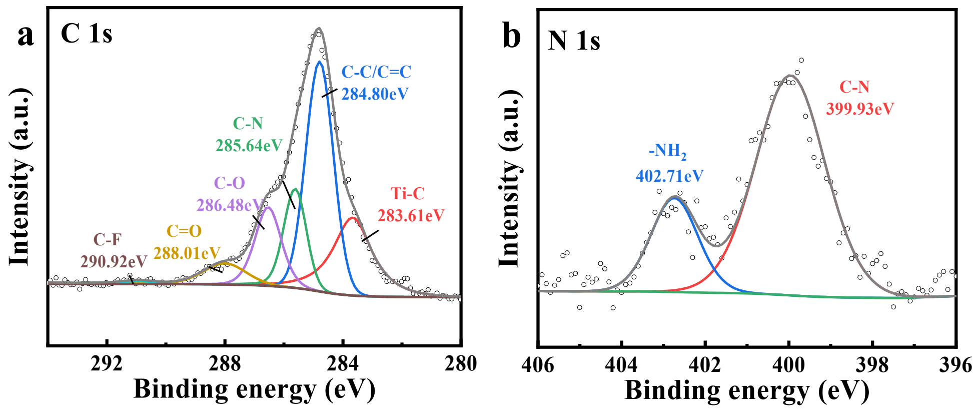

Fig. S2 XPS spectra of C1s(a) and N1s(b) for the mPM-1.

Table S1. The absorption parameters of mPM with different MXene to Py ratio

Sample	Minimum RL (matched thickness)	EBW
mPM-0.5	-17.54 (1.2 mm)	4.72 GHz (13.28-18 GHz)
mPM-1	-67.82 dB (1.37 mm)	3.68 GHz (14.16-17.84 GHz)
mPM-1.5	-19.70 dB (1.16 mm)	1.12 GHz (16.88-18 GHz)
mPM-2	-15.51 dB (3.95 mm)	1.44 GHz (15.75-17.2 GHz)

Table S2. The absorption parameters of PM with different pore sizes

Sample	Pore size	Minimum RL (matched thickness)	EBW
mPM-50	15 nm	-11.08 dB (4.5 mm)	0.12 GHz (17.88-18.00 GHz)
mPM-1	18 nm	-67.82 dB (1.37 mm)	3.44 GHz (12.88-16.32 GHz)
mPM-150	24 nm	-20.03 dB (4.0 mm)	1.58 GHz (15.12-16.70 GHz)
